# Magnetic resonance arthrography in patients with multidirectional instability: could inferior capsulsar width be considered the cornerstone in the diagnosis of non-traumatic shoulder instability?

**DOI:** 10.1007/s00256-022-04090-w

**Published:** 2022-06-30

**Authors:** Angelica Celentano, Marco Porta, Marco Calvi, Giuseppe Basile, Alberto Aliprandi, Eugenio Annibale Genovese

**Affiliations:** 1 Department of Diagnostic and Interventional Radiology, Insubria University, Varese, Italy; 2 Department of Radiology, Istituti Clinici Zucchi, Monza, Italy; 3 Department of Diagnostic and Interventional Radiology, ASST-Settelaghi, Ospedale di Circolo e Fondazione Macchi, 21100 Varese, Italy; 4 Trauma Surgery IRCCS Orthopaedic Institute Galeazzi, Milan, Italy; 5 Insubria University, Varese, 21100 Italy; 6 Clinical Medical Center - Columbus / Intermedica, Milan, 20149 Italy

**Keywords:** Inferior capsular laxity, Magnetic resonance arthrography, Multidirectional shoulder instability, Shoulder capsular redundancy

## Abstract

**Objectives:**

To provide quantitative anatomical parameters in patients with and without non-traumatic multidirectional instability using MR arthrography (MR-a).

**Materials and methods:**

One hundred and seventy-six MR-a performed from January 2020 to March 2021 were retrospectively evaluated. Patients were divided according to the presence of clinically diagnosed multidirectional shoulder instability (MDI). Each MR-a was performed immediately after intra-articular injection of 20 ml of gadolinium using the anterior approach. The width of the axillary recess, the width of the rotator interval, and the circumference of the glenoid were measured by three independent radiologists, choosing the average value of the measurements. The difference between the mean values of each of the three parameters between the two study groups was then assessed.

**Results:**

Thirty-seven patients were included in the study (20 in the MDI group, 17 in the control group). The mean axillary recess width in the MDI group was significantly greater than in the control group (*t*(33) = 3.15, *p* = .003); rotator interval width and glenoid circumference measurements were not significantly different (*t*(35) = 1.75, *p* = .08 and *t*(30) = 0,51, *p* = .6, respectively).

**Conclusions:**

Inferior capsular redundancy may be an important predisposing factor in MDI, while glenoid circumference is not related to MDI. The relationship between the width of the rotator interval and shoulder instability remains debated.

## Introduction

Shoulder macro-instability includes different clinical entities [1]. These are classified into two groups following the etiopathogenetic features and possible therapeutic options:

- Traumatic instability/traumatic etiology, unidirectional instability, Bankart lesion, surgery required (TUBS).
- Atraumatic instability/atraumatic or minor trauma, multidirectional instability, bilateral, rehabilitation, inferior capsular shift (AMBRI) [1, 2].

Multidirectional shoulder instability (MDI) is characterized by generalized instability at least in two planes of motion (anterior, posterior, or inferior) due to capsular redundancy. The features of MDI were first described by Neer and Foster in 1980 [3, 4]. Diagnosis is made clinically and strongly depends on the patient’s history: patients may present with a sulcus sign (two or more axes), positive apprehension, load and shift, and hyperabduction tests. Signs of generalized hypermobility may also be present including elbow or metacarpophalangeal joint hyperextension, genu recurvatum, patellar instability, and the ability to rest the thumb on the ipsilateral forearm, as assessed by Beighton’s criteria: if > 4/9 patient is considered hyperlax [5].

Unlike patients with traumatic shoulder instability, patients with MDI are more likely to experience episodes of recurrent dislocation [6].

Imaging may be useful in the diagnosis of MDI; in particular magnetic resonance arthrography (MR-a) may demonstrate an increased capsular volume defined by the glenocapsular ratio [1, 7], although these measures are difficult to reproduce [8].

Previous studies have demonstrated that rotator interval and axillary recess width correlate with MDI while anterior or posterior capsular redundancy shows no correlation [1, 7]. Furthermore, glenoid bone loss and version are known to be instability factors in the development of shoulder instability [9] and discrepancy in size between the small glenoid fossa and the humeral head also plays an important role [10].

The objective of our study is to demonstrate whether there is a quantitatively measurable anatomical difference between patients with and without clinically diagnosed multidirectional instability with specific attention to size of the recess at the rotator interval, inferior joint capsule recess size, and glenoid perimeter size.

## Materials and methods

### Patients

We retrospectively reviewed 176 shoulder MR-a progressively performed in the Radiology Department of our clinic from January 2020 to March 2021.

The reports of all patients examined in the given time frame were included.

The selected studies were divided into two distinct groups separating patients with suspected multidirectional instability (MDI) from patients who received MR-a for other reasons (mainly painful shoulder conditions with unclear or non-conclusive diagnosis in standard shoulder MRI).

In the study group, all patients had an atraumatic onset, and MDI of the shoulder was diagnosed by an orthopedic surgeon with 25 years of experience, based on clinical history and physical examination documenting symptomatic laxity, the presence of sulcus sign apprehension, and relocation examination.

The control group included symptomatic patients who had either a normal shoulder MR-a, tendinosis of the rotator cuff, partial-thickness tear of the rotator cuff involving less than half of the tendon thickness, or SLAP lesions.

We excluded patients with any condition which could have changed intra-articular volume such as previous surgery, extravasation of the contrast medium, full-thickness rotator cuff tear, capsular tear, bone deficiency (glenoid bone loss), or adhesive capsulitis, and every anatomical variant (i.e., Buford complex, sublabral foramen, superior sublabral recess, hypoplasia of the middle glenohumeral ligament, hypoplasia or agenesis of the superior glenohumeral ligament, hypoplasia of the glenoid labrum upstream of the glenoid notch, type III insertion of the anterior joint capsule, etc.…).

### MR arthrography imaging protocol

A 1.5-T MR imaging system (Achieva XR, Philips) was used with a dedicated shoulder array coil. The patients were placed supine with the shoulder in neutral position, the arm placed along the side, and the thumb pointing upwards. All patients were asked to give written informed consent before the procedure. MR-a was performed immediately after the intra-articular injection of 20 ml of paramagnetic contrast medium (Dotarem 2.5 mmol/l, Guerbet), using the anterior approach under ultrasound guidance (20 ml is intended as the maximum volume; in cases where resistance to injection was detected or pain appeared, the injected volume was lower). The image acquisition protocol is summarized in Table 1.

**Table 1:** MRI parameters: image acquisition protocol

Axial T1-weighted spin-echo sequences with isotropic voxel (multiplanar reconstruction) non-fat-saturated: RT (repetition time) 9.5 ms, ET (echo time): 4.7 ms, FA (flip angle): 7°, matrix 320 × 307 pixels, 0.8 × 0.8 mm pixel size, NSA (number of signal averages): 1, thickness: 0.54 mm: repetition time (RT): 500 ms, echo time (ET): 12 ms, thickness: 3.5–4 mm*	Oblique-coronal and Oblique-sagittal T1-weighted turbo spin echo (TSE T1) sequences non-fat-saturated: RT: 500 ms, ET: 18 ms, FA: 90°, matrix 384 × 307 pixels, 0.8 × 0.8 mm pixel size, NSA: 1, thickness: 3.5–4 mm*	Oblique-coronal fat-saturated PD/T2-weighted (dual) fast spin echo (FSE PD/T2 FAT SAT) sequences: RT 4000 ms, ET: 10/80 ms, FA: 90°, matrix 230 × 256 pixels, 0.8 × 0.8 mm pixel size, NSA: 1, thickness: 3.5–4 mm*

The field of view (FOV) was variable from 16 to 20 cm

### Analysis of MR images

**Fig. 1:**
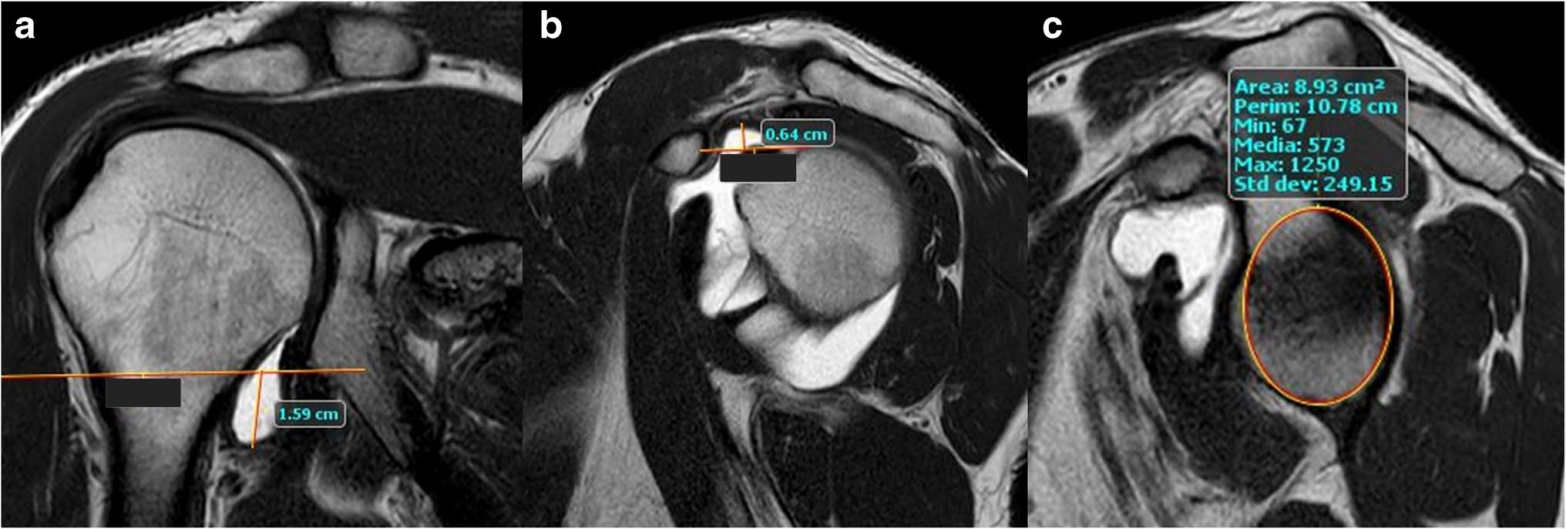
Oblique-coronal (**a**) and oblique-sagittal (**b**, **c**) T1 non-fat-sat MR-a images obtained from a 25-year-old female with non-traumatic MDI. (**a**) Axillary recess amplitude measurement method. We measured the distance between the widest point of the recess and the inferior margin of the glenoid. (**b**) Rotator interval amplitude measurement method. We considered the degree of convexity of the coracohumeral ligament relative to the line passing through the midpoint of the coracoid process and tangent to the humeral head. (**c**) Glenoid circumference measurement method. A circumference is placed at the level of the joint closely resembling the glenoid joint surface

In the selected oblique coronal and oblique sagittal T2 sequences, the following quantitative variables were measured (expressed in cm):

- Axillary recess width was measured in the oblique-coronal plane at the point of maximum amplitude, manually measuring the distance between the widest point of the recess and the inferior margin of the glenoid (Fig. 1a).
- Rotator interval width was measured in the oblique-sagittal plane, measuring the degree of convexity of the coracohumeral ligament relative to the line passing through the midpoint of the coracoid process and tangent to the humeral head at its widest point (Fig. 1b).
- The circumference of the glenoid was measured in the oblique-sagittal plane, placing the biggest circumference possible at the level of the joint closely resembling the glenoid joint surface (Fig. 1c).

The described parameters were measured on each image by three different radiologists blind to each other. During imaging evaluation, radiologists were blinded to the patient’s clinic.

### Statistical analysis

All collected data were fed into an Excel worksheet dividing the multidirectional instability group from the control group. The distribution of the data series obtained from both groups was tested using the Shapiro–Wilk test. In case of normal distribution, the Student’s *t*-test was used to determine whether there were any differences between the sample with multidirectional instability and the control group. Alternatively, the Mann–Whitney test for non-parametric continuous variables was used. Quantitative variables were presented as mean (median ± SD). It was decided to accept the results as statistically significant with *p* < 0.05.

If a significant difference is identified in one of the variables under study, the diagnostic cut-off will be calculated by considering the diagnostic performance of the variable using a ROC curve.

The data analysis for this paper was generated using the Real Statistics Resource Pack software (release 7.6, Copyright (2013–2021) Charles Zaiontz—https://www.real-statistics.com/).

## Results

**Fig. 2:**
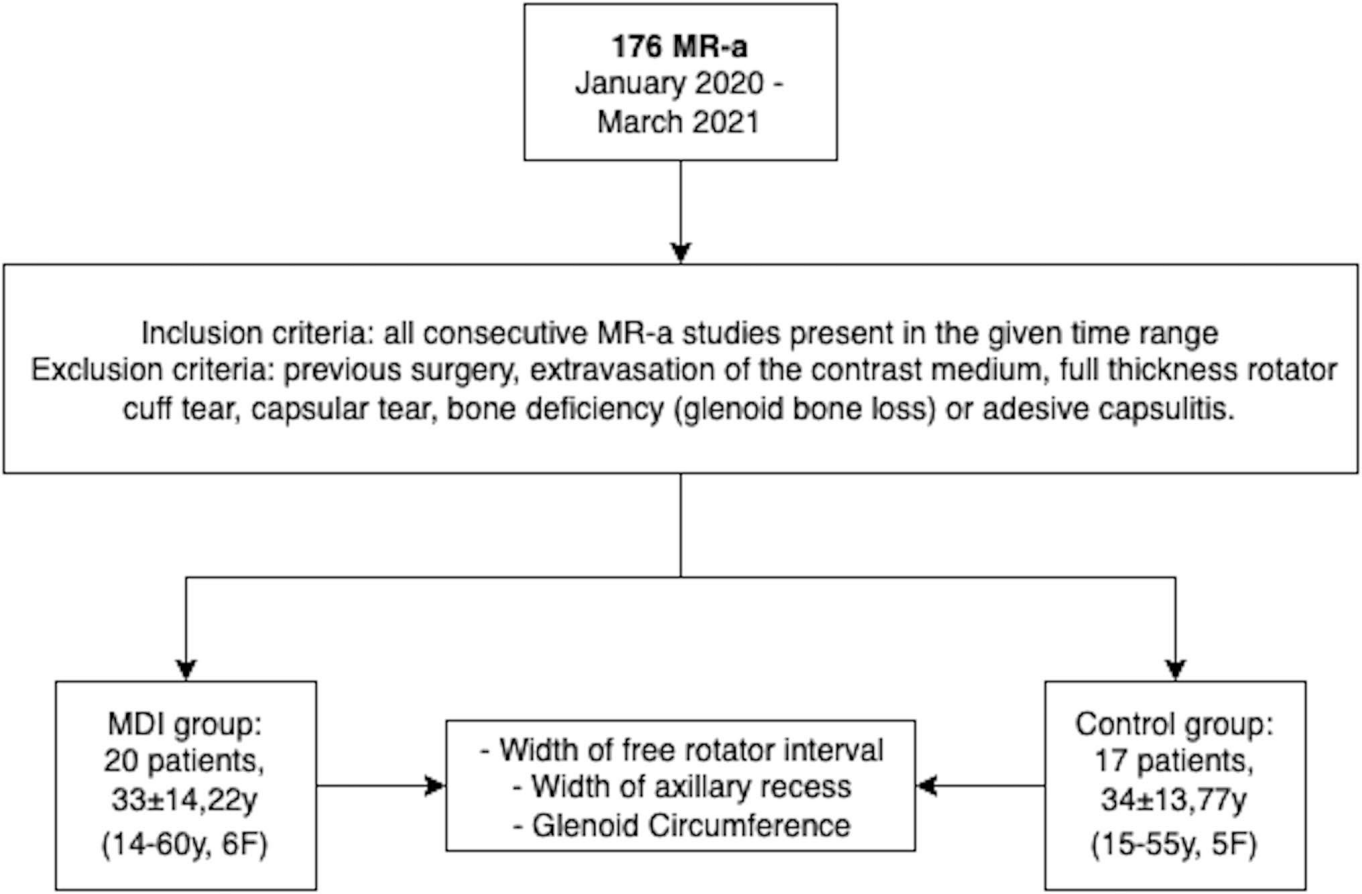
Flowchart showing the selection of records that satisfied the necessary conditions to be included in the study. MR-a, magnetic resonance arthrography; MDI, multidirectional instability

Among the retrieved examinations, after applying the exclusion criteria, 37 studies were selected as described in Fig. 2.

Of these 37 patients, 20 were clinically diagnosed with multidirectional instability, while 17 received MR-a for other reasons.

The patients’ average age was 32.68 ± 14.22 years (range: 14–60 years, 6 female) in the case group and 33.69 ± 13.77 years (range 15–55 years, 5 female) in the control group. Regardless of gender, the average measurements of axillary recess, rotator interval, and glenoid circumference in MDI patients were, respectively, 1.86 cm (1.89 ± SD 0.3), 0.8 cm (0.8 ± SD 0.2), and 7.3 cm (7.18 ± SD 1.2) (Table 2).

**Table 2:** Study results. Only the width of the axillary recess reached statistical significance

	MDI	Control group	*p*
Width of the axillary recess (cm)	1.89 ± 0.3	1.58 ± 0.3	0.003
Width of the rotator interval (cm)	0.8 ± 0.2	0.7 ± 0.18	0.08
Glenoid circumference (cm)	7.18 ± 1.2	7.86 ± 1.5	0.6

Data are mean ± standard deviation, *p* value calculated using Student’s *t*-test; *MDI*, multidirectional instability

The same measurements in the control group were 1.53 cm (1.58 ± SD 0.3), 0.6 cm (0.7 ± SD 0.18), and 7.5 cm (7.86 ± SD 1.5).

The Shapiro–Wilk test for normality proved positive in all the considered groups. Given the normal distribution of all datasets, the two groups were compared using Student’s *t*-test, which yielded the following results: the average axillary recess width in the MDI group was significantly greater than in the control group (*t*(33) = 3.15, *p* = 0.003); no significant differences were found in rotator interval width or glenoid circumference measurement (*t*(35) = 1.75, *p* = 0.08 and *t*(30) = 0.51, *p* = 0.6, respectively).

**Fig. 3:**
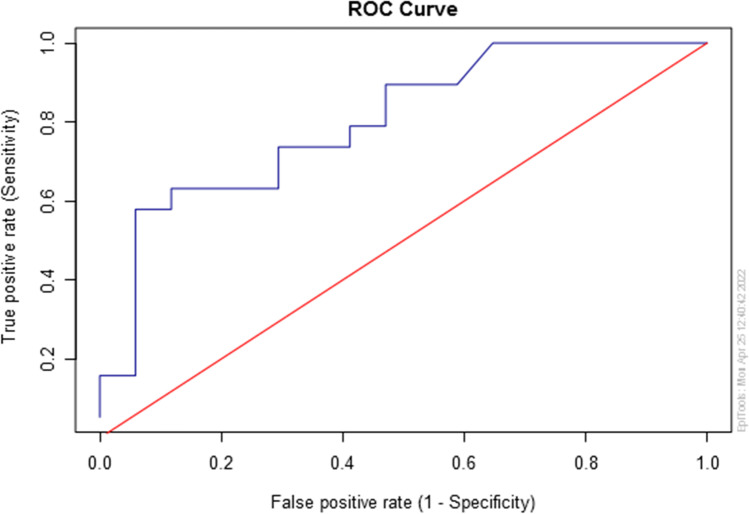
ROC curve obtained using axillary recess amplitude as the diagnostic test

Using a cut-off value of 1.89 cm, the sensitivity, specificity, and overall performance of the test based on axillary recess amplitude are respectively 0.579, 0.941, and 0.802 (95% CI 0.656–0.948) (Fig. 3).

## Discussion

Multidirectional instability of the shoulder is a complex pathology to diagnose and requires experience from the clinician. Starting from cadaveric studies, it is largely accepted that capsular redundancy is one of the key points in the development of MDI [11, 12]. MRA can provide useful information to the clinician about the actual origin of clinically evident MDI, whether due to predisposing anatomical variants, unexpected injuries, or actual capsulo-ligamentous laxity [13].

Different methods have been previously reported; some authors have shown how an increased capsular volume, expressed as the three-dimensional capsular volume with respect to glenoid surface, and an increased sagittal cross-sectional capsular area are related to MDI [8, 14]. However, they also observed that the glenoid surface area is not significantly different in patients with or without atraumatic instability. This result confirms one of the findings of our study: glenoid circumference is not significantly different between MDI patients and control group patients. These results suggest that glenoid dimension is not linked to MDI, with the exception of the presence of a bony Bankart lesion, which, instead, is strongly correlated with traumatic instability [9, 13].

The width of the rotator interval has been previously studied on both traumatic [15] and atraumatic shoulder. Patients with chronic anterior traumatic instability have been proven to have an increased rotator interval height, area, and index [13]; also, patients with multidirectional atraumatic instability have increased width and depth of rotator interval and superior capsular elongation, compared to patients without instability [12, 14, 16]. We do not find statistically significant difference in terms of rotator interval width between patients with and without atraumatic shoulder instability, in line with a previous study by Provencher et al., who found no difference of rotator interval dimension expressed as the shortest distance between the anterior edge of the supraspinatus tendon and the superior edge of the subscapularis tendon [17]. On the contrary, they found that the long head of the biceps tendon assumed a more anterior position relative to the supraspinatus tendon in patients with posterior instability. An explanation for these results might lie in the limited number of patients in both the studies, although they agreed to say that there is a relationship between MDI and rotator interval dimensions [18].

As inferior instability is the main component of MDI of the shoulder, previous authors experimented different methods to measure capsular redundancy, such as gleno-capsular ratio or labro-capsular distance and they all agree that increased axillary recess depth is correlated to shoulder instability [8, 10]. Lee et al. and Kim et al. also found significant correlation between inferior capsular redundance and MDI [19, 20]. Our results are in line with these previous studies as we observed that the width of the axillary recess at its largest point is significantly increased in patients with clinically diagnosed MDI, compared with patients without instability.

Our study has some limitations: first its retrospective nature and the limited number of patients, but MDI patients were carefully selected as well as control group patients by applying rigorous exclusion criteria, listed above.

Second, capsular volume and capacity varied from patient to patient; therefore, the amount of distention of the joint was not well controlled. However, the injection was performed by the same expert musculoskeletal radiologist to avoid another potential bias due to the variation of contrast material injection. Lastly, our results are related to patients with symptomatic instability, but not with asymptomatic hyperlaxity.

Finally, since this was a retrospective study, it was not possible to obtain an asymptomatic control group. Consequently, we do not know the measures present in completely asymptomatic patients.

In conclusion, our results confirm that axillary recess width may be used in complex clinical situations where a pattern of multidirectional instability may pre-exist or overlap with other clinical conditions. Any corrective surgery should in fact take into account the possible presence of multidirectional instability due to capsular laxity. On the contrary, glenoid circumference is not related to MDI. The relationship between rotator interval width and instability remains debated as statistical significance was not achieved in our case when comparing the two groups.

## Data Availability

The datasets used and/or analyzed during the current study are available from the corresponding author on reasonable request.

## Abbreviations

MDI: Multidirectional instability
AMBRI: Atraumatic, multidirectional, bilateral (frequently), rehabilitation (often responds to) and inferior capsular shift
TUBS: Traumatic unilateral dislocations with a Bankart lesion requiring surgery
MR-a: Magnetic resonance arthrography
MR: Magnetic resonance
